# How Shall the Stomatologist Be Educated?

**Published:** 1910-07-15

**Authors:** James E. Power

**Affiliations:** Providence, R. I.


### HOW SHALL THE STOMATOLOGIST BE EDUCATED?

JAMES E. POWER, M.D., PROVIDENCE, R. I.

In discussing the subject "How Shall the Stomatologist be Educated?" I am conscious of the fact that true science aims at the attainment of truth; honest criticism and honest discussion are the only factors that will or can assist in the accomplishment of her aim. I do not assume an attitude of criticism; I am simply responding to a call which I consider to be the duty of every member of that profession which has greater possibilities for the future than any of her sister professions, so called.

That there are branches of pathology, or I might say of pathologic physiology, which have never been explored, is a fact too obvious for discussion here. It is an accepted fact that dentists and dentistry have, in the services which they have rendered, accomplished facts which are only short of marvelous. If we read the history of dentistry and reflect on the strides which have been made in the preservation of the teeth, we may indeed point with pride to a noble record. But we must be honest with ourselves, and if we are to assist in placing this profession in that place which it should rightly occupy among the other professions we cannot content ourselves with the fact that it is so much better than it was twenty or thirty years ago; we must ask ourselves, in consideration of the number of the thousands of men and women who are going into the profession of dentistry—as well as those who have been practicing it for years, and in consideration of the number of dental colleges which exist to-day. Does this profession occupy the position that it should occupy at present? We all know the answer, and unless enthusiasm or deceit replace honest judgment and honest opinion, the answer spells, No. Now we must submit to a little self-

*Read in the Section on Stomatology of the American Medical Association, at the Sixty-first Annual Session, at St. Louis, June, 1910.

examination and determine whether or not the fault lies with us as individuals or as a profession, or whether or not we are doing the best we can with the training which we have received at our dental colleges. It is reasonable to believe that most of us are doing all we can to make our profession the greatest in the world. Then to continue to reason along logical lines, the only conclusion is that our education is confined too much to the mechanical or artistic department of our profession.

### WHAT DENTISTRY COMPREHENDS.

Before proceeding further, let us here understand whether we consider that dentistry comprises merely the making of artificial dentures, extracting teeth and insertion of fillings, or whether it comprehends something more; for instance, whether it has a direct relation to histology and physiology; whether it should have a part in the interpretation of physiologic and pathologic metabolism; whether it should have a complete knowledge of those factors which preserve health and breed disease, not only of the teeth, but of the mucous membranes from the mouth through the whole alimentary canal, with all its adjacent and component parts? If we accept the former definition, then I say that we are highly trained technicians, and yet not as highly trained or as well developed in the manipulative skill as are some of our colaborers, who are not considered by us to belong to a profession at all.

If we accept the latter, then I say that our education is inadequate, and that in order to fit ourselves to fulfill the obligations which are expected of us, and to make our profession all that our fathers intended it should be, we are obliged to call for a change in the teaching of the future students of dentistry. Let us agree to stop asking for recognition, from a profession which many of us believe is only to be our equal, namely, the medical profession. There never was and there never will be either an individual or a profession that need call for recognition. Recognition is that indefinable something which is always thrust on the individual when that individual occupies the position which is deserving of such recognition. The world is the judge and the world gives the recognition as soon as we deserve it. No sooner, no later.

### FOUNDATIONS IN DENTISTRY AND MEDICINE.

That the foundation on which the dental graduate is sent into the world is greatly inferior to the corresponding foundation of the graduate in medicine is a well-known fact to all who have taken the time and patience to investigate the educational advantages of each. The dental graduate will tell you that he studied the same subjects as did the medical student. This is true, but the question is, Did he study them as thoroughly, or rather, was the standard for him and his medical brethren equal? In a large majority of cases he is laboring under a delusion if he believes that he knows as much, or even if he thinks he was given the same advantages in learning pathology, bacteriology, biological chemistry, or even anatomy, or physiology as was his medical brother. And now let me anticipate your question. Why? Because this branch of the science is taught by the professors of medicine, and in some instances the members of the dental faculty, who were graduated twenty-five or thirty years ago,, do not seem to understand the importance of a proper medical education. We are apt to become subjects, if not indeed victims, of our environment, and the dental student is no exception to the rule. He breathes an atmosphere while on the dental side of the college, which may give his mind a bias. This condition of mind is not always correct, and when the student comes under the instruction of the professors of medicine, the men who are spending their lives in fitting the candidate for dental honors (for that seems to me the most important part of their life's work), the mind of the dental student is apt to be antagonistic to their teachings. If the mind was directed by the dental educators (the men who first meet the students) along the proper channels, it would be receptive and instead of leaving these departments with a superficial, or I might say an artificial knowledge, they would have a foundation on which they could and would develop the future of our profession.

### WHAT THE COURSE SHOULD COMPRISE.

I am firmly convinced that a complete medical education is absolutely necessary for the proper practice of what the profession of dentistry means to me. In replying to the first question, "When is it best to begin the special studies; during the time of the general medical studies or immediately after these?" I will state that the studies of the dental specialist should be postponed until after the medical education is completed. I do not belive that there should be introduced into the medical course any of the special studies of the dentist except those which are already recognized as belonging to the department of medicine. If we introduce any of the special studies during this time we are destroying that which we are trying to accomplish, a complete medical training and the mind of the student is again diverted from the direct pathway. The special studies of the dentist should commence after his medical education is completed and should consist of certain branches of prosthetic dentistry, certain parts of crown and bridgework, the fundamental principles which govern the correction of irregularities of the teeth, and oral surgery. One year should be sufficient for the above named studies. The operative dentistry should consist of a practical teaching of filling teeth, and a theoretical teaching of the composition of the various filling materials. The prosthetic dentistry should consist of how to make artificial dentures, and a theoretical knowledge of the material, eliminating all the antiquated methods of making artificial dentures, leaving this for the dental historians. The crown and bridgework course should be carried along the same lines as the prosthetic course, viz., eliminating the antiquated methods of doing this work, as well as its history.

Have men in charge in these departments who can and will teach the short methods of doing this work. Orthodontia can be taught as it is taught now. I am confident that one year would be sufficient for the proper teaching, as well as the proper understanding of the special studies required of the dentist. I realize that some may say it is unreasonable to believe that one year is sufficient to teach that which at the present time takes three years to teach. In replying I will state that there must be a revolution in the present teaching, and with a medical education the student has already learned all the material, which is now but poorly taught in the dental courses. His mind is more highly developed, as well as his powers of interpretation, not only of pathological, but of all conditions pertaining to the practice of a specialty, and the time required for a proper understanding would, of course, be shorter.

To me it seems impossible that a person can learn pathology without a microscopic study of all diseased tissue. How can we understand bacteriology without studying micro-organisms, both in culture media and under the lens of the microscope? How can we understand the subject of immunity and apply the vaccines and sera to the diseases which come under our specialty if we do not understand thoroughly that subject which is already revolutionizing the treatment of disease? There is not in reality, and there should not be in name, dental pathology, dental materia medica, dental neurology and dental hygiene, and numerous other dental subjects which are really a part of the great subjects themselves and are included in them. It is agreed that a great deal of time is wasted in the attempt to teach these subjects, and the shame of it all is that they are taught imperfectly. If we eliminate these subjects and revise the teaching of the dental subjects, then we will have the same amount of time for the dental subjects as we have now, only we have a better foundation on which to base this work, which at most could be accomplished in one year.

### THE DAILY SCHEDULE.

Saturday morning could be devoted to the orthodontia clinic and Saturday afternoon to the oral surgery, this branch including the extraction of teeth; Monday morning to be devoted to the crown and bridgework clinic; every other morning to be devoted to the practical prosthetic work, and every afternoon, Saturday excepted, to the teaching of practical operative dentistry (filling of teeth, etc.). The treatment of the orthodontia patients subsequent to the visit at the clinic Saturday morning could be carried on during the latter part of the afternoon of the operating days, because it requires in the vast majority of cases, but a few minutes work. The lecture on operative dentistry, crown and bridgework, prosthetic dentistry, and orthodontia could be given at the first morning exercise, which could be easily arranged at 8:30. Commence the practical work at 9:30 and continue it till 1 o'clock. Resume the afternoon studies at 2 and continue till 6 o'clock.

### CONCLUSIONS.

Let us make up our minds to have more of the real, and less of the make-believe. Let us agree on the position we are assuming. If we are highly trained technicians, then reduce the hours of study by eliminating the make- believe part of our course. If we are highly trained scientific physicians of special diseases, then let us substitute the make- believe for the real; let us pursue the same course as do other specialists. Why hesitate to add our names to the honor roll, to follow the footsteps of the men who have already spent more time than I have outlined to get that medical education which seems an essential equipment for us to explore those regions which are now darkened by ignorance. The value of the medical education is already proved by the fact that most of the truly scientific discoveries are made by the men and women who have supplemented their dental knowledge with a medical education.

In conclusion let me add those words of Hunter which inspired Jenner to make one of the greatest discoveries in modern medicine, namely, vaccination: "Don't think; but try; be patient; be accurate."—*Journal A. M. A.*, June 25th, 1910.

